# *PpYUC11*, a strong candidate gene for the stony hard phenotype in peach (*Prunus persica* L. Batsch), participates in IAA biosynthesis during fruit ripening

**DOI:** 10.1093/jxb/erv400

**Published:** 2015-08-24

**Authors:** Lei Pan, Wenfang Zeng, Liang Niu, Zhenhua Lu, Hui Liu, Guochao Cui, Yunqin Zhu, Jinfang Chu, Weiping Li, Weichao Fang, Zuguo Cai, Guohuai Li, Zhiqiang Wang

**Affiliations:** ^1^Key Laboratory of Fruit Breeding Technology, Ministry of Agriculture, Zhengzhou Fruit Research Institute, Chinese Academy of Agricultural Sciences, Zhengzhou 450009, China; ^2^Key Laboratory of Horticultural Plant Biology, Ministry of Education, Huazhong Agricultural University, Wuhan 430070, China; ^3^National Centre for Plant Gene Research, Institute of Genetics and Developmental Biology, Chinese Academy of Sciences, Beijing 100101, China

**Keywords:** Auxin, ethylene, microsatellite, *Prunus persica* L. Batsch, ripening, stony hard, YUCCA flavin monooxygenase.

## Abstract

High concentrations of indole-3-acetic acid (IAA) are required for climacteric ethylene biosynthesis to cause fruit softening in melting flesh peaches at the late ripening stage. By contrast, the fruits of stony hard peach cultivars do not soften and produce little ethylene due to the low IAA concentrations. To investigate the regulation of IAA accumulation during peach ripening [the transition from stage S3 to stage S4 III (climacteric)], a digital gene expression (DGE) analysis was performed. The expression patterns of auxin-homeostasis-related genes were compared in fruits of the melting flesh peach ‘Goldhoney 3’ and the stony hard flesh peach ‘Yumyeong’ during the ripening stage. It is revealed here that a YUCCA flavin mono-oxygenase gene (*PpYUC11*, ppa008176m), a key gene in auxin biosynthesis, displayed an identical differential expression profile to the profiles of IAA accumulation and *PpACS1* transcription: the mRNA transcripts increased at the late ripening stage in melting flesh peaches but were below the limit of detection in mature fruits of stony hard peaches. In addition, the strong association between intron TC microsatellite genotypes of *PpYUC11* and the flesh texture (normal or stony hard) is described in 43 peach varieties, indicating that this locus may be responsible for the stony hard phenotype in peach. These findings support the hypothesis that *PpYUC11* may play an essential role in auxin biosynthesis during peach fruit ripening and is a candidate gene for the control of the stony hard phenotype in peach.

## Highlight

*PpYUC11* is a strong candidate gene for the control of the stony hard phenotype in peach.

## Introduction

Peach (*Prunus persica* L. Batsch) is a climacteric fruit in which the burst of ethylene production occurs at the ripening stage (Tonutti *et al.*, 1991, 1997). Peach fruits generally soften rapidly after harvest, causing difficulties in postharvest handling and quality maintenance during storage and transportation (Yoshioka *et al.*, 2010). Improving the fruit texture quality of peaches is one of the most important goals of cultivar development and selection, superseded only by appearance and harvest season (Gallardo *et al.*, 2012). Based on the characteristics of fruit firmness and textural changes during ripening, peach cultivars can be classified into three groups: melting (M), non-melting (NM), and stony hard (SH) flesh types (Bailey and French, 1932; Yoshida, 1976; Haji *et al.*, 2005). The M phenotype shows a prominent softening at advanced stages of ripening and develops a complete melting texture, whereas NM-textured peaches soften slowly when overripe and never melt. Peaches of the SH type, the focus of the present study, have very firm and crisp flesh at ripening (both on- and off-tree), although they change colour normally and contain a high level of soluble solids (Haji *et al*., 2001, 2004).

Endopolygalacturonase (endo-PG), the enzyme responsible for cleaving polygalacturonic acid chains (pectins) from the cell wall, is thought to be responsible for the M/NM flesh texture (Lester *et al.*, 1996; Callahan *et al.*, 2004). This trait is controlled by the ‘*melting*’ locus, where the *melting* (*M*) is dominant over the *non-melting* (*m*) (Bailey and French, 1932). A high level of ethylene production (in the late ripening phase) characterizes the M and NM varieties. This phase, however, is absent in the SH phenotype which is thought to contribute to the inhibition of fruit softening in stony hard peaches because exogenous ethylene softens the flesh effectively; thus, the fruit softens more rapidly when a higher concentration of ethylene is applied (Haji *et al.*, 2003; Hayama *et al*., 2003, 2006). Treatment of SH fruits with 1-aminocyclopropane-1-carboxylic acid (ACC), the immediate precursor of ethylene, stimulated the fruits to synthesize ethylene and to soften (Haji *et al.*, 2003). These results indicate that a low activity of ACC synthase is responsible for the inhibition of ethylene production and that ACC oxidase and the ethylene receptor function normally in SH peaches (Haji *et al.*, 2003). Genetic analysis suggested that the *Stony hard* trait is controlled by a single recessive gene (*hd*) (Yoshida, 1976) and is inherited independently of the *M*/*m* flesh locus (Haji *et al.*, 2005).

It has been assumed that transcription suppression of *PpACS1* in the SH phenotype is responsible for its low ethylene production (Tatsuki *et al.*, 2006); however, mRNA transcripts of *PpACS1* have been detected in senescing flowers and wounded leaves as well as in immature fruits and cold-treated mature fruits of the SH phenotype (Tatsuki *et al.*, 2006; Begheldo *et al.*, 2008). These results indicate that stress conditions (wounding or low temperature) seem to be effective in overcoming *PpACS1* inhibition that has been ascribed in SH peaches to a disruption of a transcriptional factor specifically activated at ripening (Tatsuki *et al.*, 2006). A recent study suggested that the low IAA concentrations in SH peaches may contribute to the suppression of *PpACS1* because the application of exogenous 1-naphthalene acetic acid (NAA), a synthetic auxin, to stony hard peaches induced a high level of *PpACS1* expression, the production of a large amount of ethylene, and softening (Tatsuki *et al.*, 2013).

Multiple mechanisms regulating auxin-homeostasis have been recognized in plant organs, such as auxin metabolism (biosynthesis and conjugation) and carrier-dependent intercellular and intracellular auxin transport (Rosquete *et al.*, 2012). In many cases, *de novo* IAA biosynthesis, primarily from tryptophan, is the most important source of free auxin in the plants. Genetic and biochemical studies have revealed that, in *Arabidopsis* and maize, auxin biosynthesis is controlled by a two-step pathway: tryptophan is first converted into indole-3-pyruvate (IPA) by the TAA family of aminotransferases, and, subsequently, IAA is produced from IPA by the YUCCA family of flavin monooxygenases (Mashiguchi *et al.*, 2011). In addition to *de novo* synthesis, plants produce active IAA by hydrolysing IAA conjugates. Three major forms of auxin conjugates exist in higher plants, including IAA–amino acid conjugates, IAA–sugar conjugates, and IAA–peptide conjugates (Ludwig-Müller, 2011). Group II of the GRETCHEN HAGEN3 (GH3) family facilitates IAA to amino acids (Staswick *et al.*, 2005) whereas the IAA-LEUCINE RESISTANT 1 (ILR1)-like family of amidohydrolases hydrolyse IAA–amino acid conjugates to free IAA (Bartel and Fink, 1995; Davies *et al.*, 1999; LeClere *et al.*, 2002). Our knowledge of the metabolism of IAA–sugar conjugates and IAA–peptide conjugates is far less detailed than our knowledge of the metabolism of IAA–amino acid conjugates. UDP glucosyltransferases, such as UGT84B1 in *Arabidopsis* (Jackson *et al.*, 2001) conjugate IAA to glucose. The conversion back to IAA of the presumed storage form of auxin, IBA, is catalysed by the action of peroxisomal β-oxidation enzymes, the IBRs (INDOLE-3-BUTYRIC ACID RESPONSE) (Zolman *et al.*, 2008). In addition, the spatio-temporal distribution of auxin depends on cell-to-cell auxin transport. A few studies have demonstrated that AUXIN RESISTANT 1 (AUX1)-mediated auxin influx and PIN-FORMED 1 (PIN1)-mediated auxin efflux played a key role in the regulation of organ-level auxin transport (Bennett *et al.*, 1996; Gälweiler *et al.*, 1998; Müller *et al.*, 1998; Li *et al.* 2011; Friml *et al*., 2002a, b). The low IAA concentration at the late-ripening stage of stony hard peaches would be determined by the result of one or the interaction of more mechanisms above (see Supplementary Fig. S1 at *JXB* online).

The above introduction indicates that auxins are involved in peach softening and that stony hard peach cultivars do not soften due to low IAA concentrations. To date, the molecular mechanism underlying this phenomenon has remained a mystery. Recently, digital gene expression profiling was conducted using deep-sequencing in melting/stony hard flesh peaches, and the results provide opportunities for investigating genes involved in IAA concentrations during peach ripening. It is reported that one gene in particular, *PpYUC11*, appears to be an excellent candidate gene for the stony hard phenotype in peaches, and it is proposed that the intronic TC microsatellite sequence variant controls the expression of this gene. Our study is not only helpful for understanding the molecular mechanism of IAA accumulation during peach ripening but also useful for the marker-assisted breeding of new peach cultivars with better preservability.

## Materials and methods

### Plant materials, sample collection, and NAA treatments

Thirty-six accessions of normal-textured (melting or non-melting) peaches (‘Azumo’, ‘Bai Hua’, ‘Bao Lu’, ‘Chinese Cling’, ‘CN 13’, ‘CN 5’, ‘CN 8’, ‘CN 9’, ‘CP 5’, ‘Elberta’, ‘Goldhoney 1’, ‘Goldhoney 3’, ‘Hakuho’, ‘Hakuto’, ‘Hangzhou Zao Shui Mi’, ‘Kawanakajima Hakuto’, ‘Li Chun’, ‘Matsumori’, ‘May fire’, ‘NJC105’, ‘NJC112’, ‘NJC19’, ‘NJC47’, ‘NJC77’, ‘Okitsu’, ‘Okubo’, ‘Pan Tao Huang Hou’, ‘Spring Snow’, ‘Springtime’, ‘Sunago Wase’, ‘Toobo’, ‘Yu Hua Lu’, ‘Zao Feng Wang’, ‘Zao Lu Pan Tao’, ‘Zhong Pan Tao 1’, and ‘Zhong Pan Tao 2’) and seven accessions of stony hard cultivars (‘CN 16’, ‘Hua Yu’, ‘Jing Yu’, ‘Qing Wang’, ‘Shi Jia Zhuang’, ‘Xia Cui’, and ‘Yumyeong’) were analysed in this study. All peach accessions were grown at the experimental farm of the National Fruit Tree Germplasm Repository, Zhengzhou Fruit Research Institute, Chinese Academy of Agricultural Sciences (Zhengzhou, China). Leaves, stems, and stem tips of ‘Goldhoney 3’ at the vegetative growth stage as well as flowers at the full bloom stage were collected during the spring of 2014. Fruit growth stages were defined according to Tonutti *et al.* (1997) and Gabotti *et al.* (2015). At the desired times (from the S3 to the S4 stage at eight time points), fruits of ‘Goldhoney 3’ and ‘Yumyeong’ were picked and quickly transferred to the laboratory for the determination of weight and ripening parameters (ethylene production, flesh firmness, and soluble solids content). Fruit mesocarp tissues and seeds (only for ‘Goldhoney 3’) at 106, 110, 115, 122, 127, 131, 134, and 140 days after full bloom (DAFB) were sampled. The auxin treatment was performed by dipping mature ‘Yumyeong’ whole fruits into 1mM NAA (1-naphthalene acetic acid) mixed with 500 μl l^–1^ Tween 20 for 15min; subsequently, the fruits were sprayed with the NAA solution every day over a period of 4 d. The control was treated with an aqueous solution of 500 μl l^–1^ Tween 20. All samples were immediately frozen in liquid nitrogen and then stored at –80 °C for DNA or RNA extraction.

### Ethylene production, flesh firmness, and soluble solids content (SSC)

Ethylene production and flesh firmness were measured as described previously (Zeng *et al.*, 2015). After the measurement flesh firmness, part of the mesocarp was squeezed and SSC was measured with an Atago digital refractometer (Atago, Tokyo, Japan).

### Extraction and UPLC-MS/MS analysis of IAA in the peach mesocarp

Extraction and purification of IAA in mesocarp samples were performed according to a previously described method (Zourelidou *et al.*, 2009, Tatsuki *et al.*, 2013), with minor modifications.

To determine the IAA content in each sample, the UPLC-MS/MS analyses were carried out as previously described by Fu *et al.* (2012).

### RNA extraction and quantitative real-time PCR

To validate the differential expression identified by DGE-seq or to detect the expression of other genes, quantitative reverse-transcription PCR (qRT-PCR) was performed as described previously (Zeng *et al.*, 2015). Eleven auxin-homeostasis-related genes with different expression patterns during the late ripening stage between the normal and stony hard flesh phenotypes were chosen for validation and the specific primers used to quantify gene expression levels are listed in Supplementary Table S1 at JXB online. The housekeeping genes have been described by Tatsuki *et al.* (2013). All gene expression analyses were performed with three independent biological replicates.

### Phylogenetic analysis

The amino acid sequences of the YUCCA family of flavin monooxygenases from *Prunus persica* and *Arabidopsis thaliana* were used for phylogenetic analysis. Multiple sequence alignment was carried out using ClustalX version 1.83 (Thompson *et al.*, 1997) and adjusted manually as necessary and the phylogenetic tree was generated by the Neighbor–Joining method using MEGA version 5 (Tamura *et al.*, 2011). Bootstrap values were calculated from 1 000-replicate analyses.

### Digital gene expression profiling (DGE) library preparation, Illumina RNA-sequencing, and data processing

Total RNA was extracted using a Plant Total RNA Isolation Kit (Sangon, China) according to the manufacturer’s instructions. RNA concentrations were measured using a NanoPhotometer spectrophotometer (IMPLEN, CA, USA), and the integrity was confirmed using the RNA Nano 6000 Assay Kit of the Bioanalyzer 2100 system (Agilent Technologies, CA, USA). DGE libraries were generated using the NEBNext Ultra RNA Library Prep Kit for Illumina (NEB, USA) following the manufacturer’s recommendations and index codes were added to attribute sequences to each sample. Each library’s quality was then assessed on the Agilent Bioanalyzer 2100 system. The library preparations were sequenced on an Illumina HiSeq 2000 platform. For gene expression analysis, the number of expressed tags was calculated and then normalized to RPKM (Reads Per Kilobase of exon model per Million mapped reads) (Mortazavi *et al.*, 2008).

### Retrieval of auxin-homeostasis-related gene sequences

Auxin-homeostasis-related gene sequences of *Arabidopsis thaliana* were identified from the *Arabidopsis* Information Resource (TAIR, https://www.arabidopsis.org/, last accessed: 18 August 2015) and auxin-homeostasis-related gene sequences of peach were obtained through the retrieval of *Prunus persica* v1.0 assembly at the Genome Database for Rosaceae (Verde *et al.*, 2013) (GDR, http://www.rosaceae.org/, last accessed: 18 August 2015) or by BLASTP search at the National Center for Biotechnology (http://www.ncbi.nlm.nih.gov/blast/, last accessed: 18 August 2015). All sequences and their accession numbers are provided in Supplementary Table S3 at *JXB* online.

### Detection of intronic TC microsatellite variations

Genomic DNA was extracted using the modified CTAB procedure (Cheng *et al.*, 1997). The concentration of DNA was determined with a NanoDrop 1000 spectrophotometer (Thermo Scientific, Wilmington, DE, USA) and diluted to 25ng μl^–1^ to carry out PCR amplification reactions. The DNA samples were then used as PCR templates for microsatellite region amplification. The intronic TC microsatellites were amplified using primers: 5′-CTATCTGGTATATAAGCTGAAACG-3′, 5′-ACCTTTTT AGTTATTTCACCACAG-3′. The PCR reactions were performed using KOD-Plus-DNA polymerase (Toyobo, Osaka, Japan) according to the manufacturer’s instructions. The DNA amplification products were loaded on to 10% polyacrylamide gels. The gels were silver stained according to the protocol described by Bassam *et al.* (1991).

## Results

### Fruit phenotype during ripening: ethylene production, flesh firmness, and soluble solids content

**Fig. 1.**
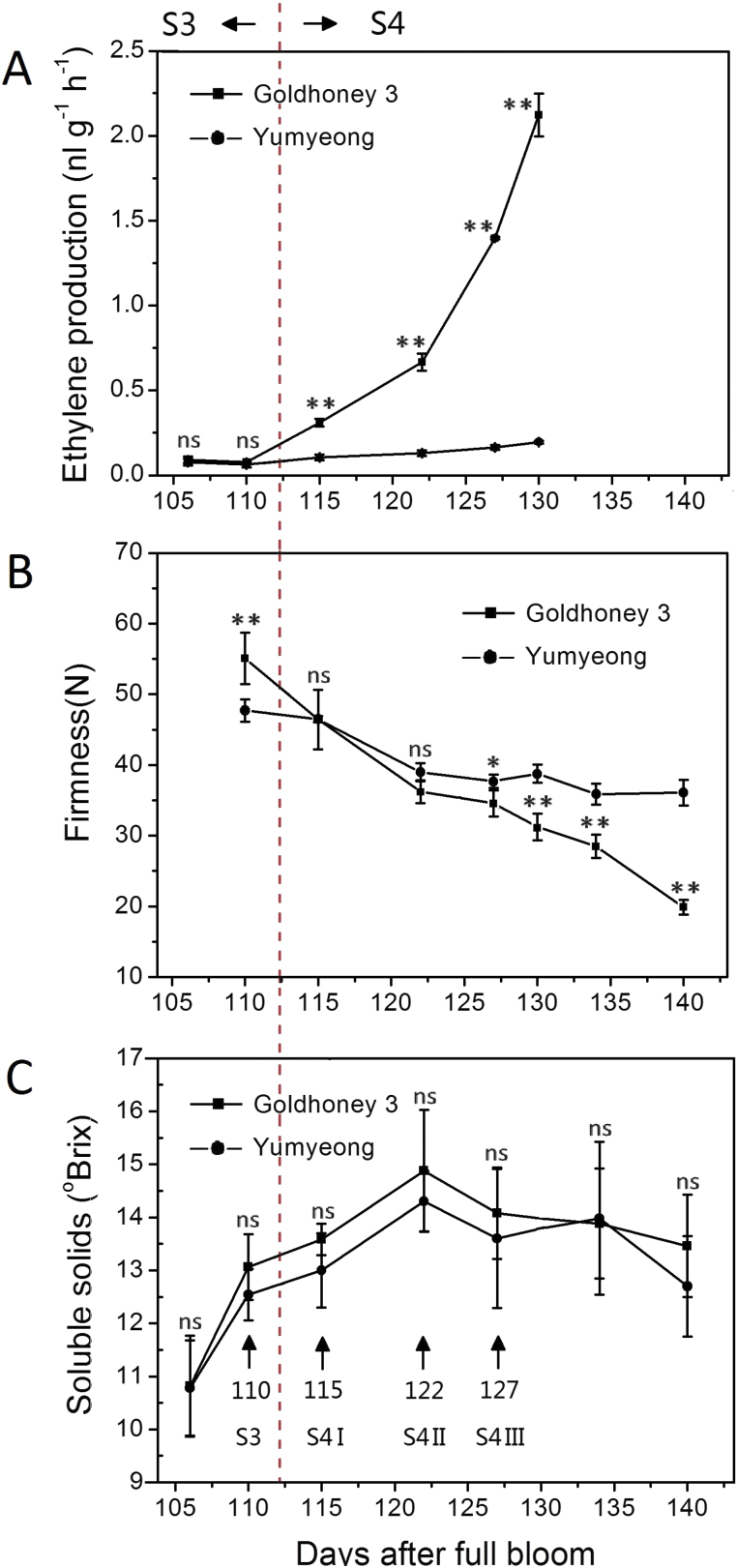
(A) Ethylene production, (B) flesh firmness, and (C) SSC of melting cultivar ‘Goldhoney 3’ and stony hard cultivar ‘Yumyeong’ fruit from the late stage of the second exponential growth phase (S3) to the climacteric stage (S4). As shown in (C), the S3, S4 I, S4 II, and S4 III time points, corresponding to 110, 115, 122, and 127 DAFB, respectively, were selected for further study. For ethylene production, the results are the mean ±SE of measurements for at least three individual experiments; for flesh firmness or SSC, the results are the mean ±SE of measurements for at least five fruits. Asterisks indicate statistically significant differences compared with ‘Yumyeong’ at a similar stage in development (days after full bloom) using Student’s *t* test (*P <0.05, **P <0.01). ns indicates that there were no significant differences compared with ‘Yumyeong’. (This figure is available in colour at *JXB* online.)

*Prunus persica* fruits of ‘Goldhoney 3’ and ‘Yumyeong’ cultivars were picked at the desired ripening stages: transition from stage S3 (second exponential growth phase) to stage S4 (climacteric). The two cultivars have different fruit flesh texture: ‘Goldhoney 3’ is a melting type that softens rapidly during ripening, and ‘Yumyeong’ is a stony hard type that remains firm during ripening. Fruits from the end of stage S3 to stage S4 were measured for ethylene production, flesh firmness, and soluble solids content (°Brix, an approximate measure of the mass ratio of dissolved solids, mostly sucrose, to water in fruit juices) (Fig. 1). Ethylene production was low in ‘Goldhoney 3’ fruit before 110 DAFB (stage S3) and it sharply increased upon reaching stage S4 whereas, in ‘Yumyeong’, ethylene production was sustained at a low level during the same period (Fig. 1A). Flesh firmness decreased substantially in ‘Goldhoney 3’ between 110 and 130 DAFB, but it decreased moderately in ‘Yumyeong’ during the same period (Fig. 1B). In ‘Goldhoney 3’ and ‘Yumyeong’, SSC was 11 °Brix at 105 DAFB; increased gradually to a peak value of 15 or 14 °Brix at 122 DAFB for the two cultivars, respectively; and then gradually decreased; the maximum SSC attained in ‘Goldhoney 3’ was higher than that of ‘Yumyeong’ (Fig. 1C). The similarity of these SSC time series is consistent with the fact that both cultivars have an identical maturity stage in Zhengzhou.

Depending on phenotypic parameters, four sampling points covering the climacteric stage at 110, 115, 122, and 127 DAFB (named S3, S4 I, S4 II, and S4 III, respectively, and marked with arrows in Fig. 1C) were selected for IAA concentration and digital gene expression (DGE) analysis.

### IAA concentration and digital gene expression (DGE) analysis of auxin-homeostasis-related genes

**Fig. 2.**
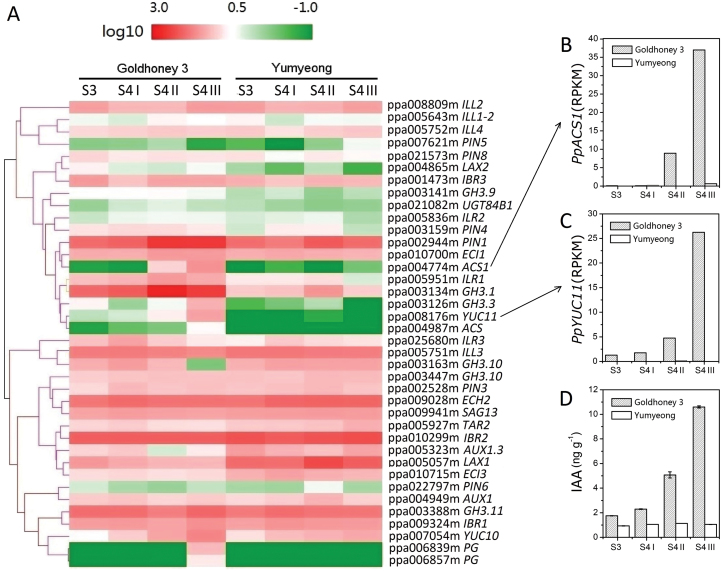
Hierarchical cluster analysis of auxin-homeostasis-related genes in peach undergoing ripening. (A) Hierarchical cluster analysis of 34 auxin-homeostasis-related genes and four ripening-related gene expression levels in ‘Goldhoney 3’ and ‘Yumyeong’; peach fruits were sampled at the S3, S4 I, S4 II, and S4 III stages. The log_10_ ratios and scale bars are shown in the resulting tree figure, which was obtained using the CLUSTER software package and Java Treeview. The DGE profiles of these genes are listed in Supplementary Table S3 at *JXB* online. (B) RPKM of *PpACS1*, (C) RPKM of *PpYUC11*, and (D) IAA concentrations in ‘Goldhoney 3’ and ‘Yumyeong’. Data are means ±SE of three individual experiments, each performed in triplicate. (This figure is available in colour at *JXB* online.)

Previous studies have shown that IAA levels significantly increased in a melting flesh cultivar at the climacteric stage, although the IAA concentration in the mesocarp tissue of a stony hard peach cultivar were low and did not increase (Tatsuki *et al.*, 2013). Before the expression patterns of auxin-homeostasis-related genes in both cultivars during fruit ripening were analysed, IAA concentrations were measured with the same samples at stages S3, S4 I, S4 II, and S4 III in ‘Goldhoney 3’ and ‘Yumyeong’ (Fig. 1C). Whereas in ‘Goldhoney 3’ fruits, the IAA levels sharply increased from stage S3 to stage S4 III, the IAA levels did not increase, and the final IAA concentration was 1.049ng g^–1^ fresh weight in ‘Yumyeong’ fruits (Fig. 2D).

To obtain the expression profiles of auxin-homeostasis-related genes for the melting peach ‘Goldhoney 3’ and the stony hard peach ‘Yumyeong’ during the late ripening stages, total RNAs were isolated from ‘Goldhoney 3’ and ‘Yumyeong’ mesocarps at stages S3, S4 I, S4 II, and S4 III (Fig. 1C). Differential gene expression profiling was performed using high-throughput Tag-seq analysis to examine the auxin-homeostasis-related gene profiles. DGE sequencing analyses of ‘Goldhoney 3’ and ‘Yumyeong’ samples were performed on the Illumina HiSeq™ 2000 sequencing platform. ‘Goldhoney 3’ (S3, S4 I, S4 II, and S4 III) and ‘Yumyeong’ (S3, S4 I, S4 II, and S4 III) produced 7 111 569, 8 765 279, 8 115 631, 8 477 817, 8 991 034, 8 839 334, 8 479 265, and 9 126 943 clean reads, respectively. The read count data were used to analyse the differences in gene expression between the two cultivars. To estimate the gene expression levels, the read counts were transformed into RPKM (Mortazavi *et al.*, 2008); RPKM >1 is defined as the threshold of significant gene expression. The full list of normalized expression of 28 702 peach genes is shown in Supplementary Table 2 at *JXB* online.

In total, 51 auxin-homeostasis-related genes were identified from the peach genome: 12 auxin biosynthesis genes (9 *YUC* genes and 3 *TAA* genes), 27 auxin conjugation genes (9 *ILL* genes, 8 *GH3* genes, 3 *UGT* genes, and 7 *IBR* genes), and 12 auxin transport genes (8 *PIN* genes and 4 *AUX1* genes). Based on our DGE data, 34 genes out of 51 total auxin-homeostasis-related genes have significant gene expression (RPKM >1) in at least one sampling point (Fig. 2A; see Supplementary Table S3 at *JXB* online), constituting the subset of candidate genes involved in controlling IAA concentration in peach fruit during the ripening stage. To identify the gene or genes putatively involved in IAA accumulation during melting peach ripening, the expression profiles of these auxin-homeostasis-related genes were compared with two peach *ACS* and two endopolygalacturonase (endo-PG) genes by DGE (Fig. 2A). In ‘Goldhoney 3’, the expression level of ACC synthase gene *PpACS1* (ppa004774m) increased towards harvest time along with other ripening-related genes [ppa006839m (endo-PG); ppa006857m (endo-PG)]. By contrast, the expression level of *PpACS1* in the stony hard peach stayed low and did not increase during ripening (Fig. 2A, B; see Supplementary Table S3at *JXB* online). A total of 34 auxin-homeostasis-related genes were significantly expressed and their expression profiles were hierarchically clustered with that of *PpACS1* and endo-PG (Fig. 2A). Interestingly, six auxin-homeostasis-related genes were found to cluster closely with *PpACS1*: one *PIN* gene (ppa002944), one *IBR* gene (ppa10700m), one *ILL* gene (ppa005951m), two *GH3* genes (ppa003134m and ppa003126m), and one *YUC* gene (ppa008176m) (Fig. 2A). Among these, two genes—the *YUC* gene (ppa008176m) and one of the *GH3* genes (ppa003126m)—showed an identical expression pattern to *PpACS1* for both cultivars (Fig. 2C). Taking the gene functions into account, the *YUC* gene (ppa008176m) may regulate IAA concentration during the fruit ripening stage of the melting flesh peach ‘Goldhoney 3’.

To confirm the gene expression changes observed by DGE analysis, quantitative PCR analysis was performed on a selection of differentially expressed auxin-homeostasis-related genes. Overall, the quantification of 11 auxin-homeostasis-related genes by qRT-PCR exhibited close agreement with DGE-seq results (see Supplementary Fig. S2 at *JXB* online).

### The effects of NAA on the expression of auxin-homeostasis-related genes in ‘Yumyeong’

**Fig. 3.**
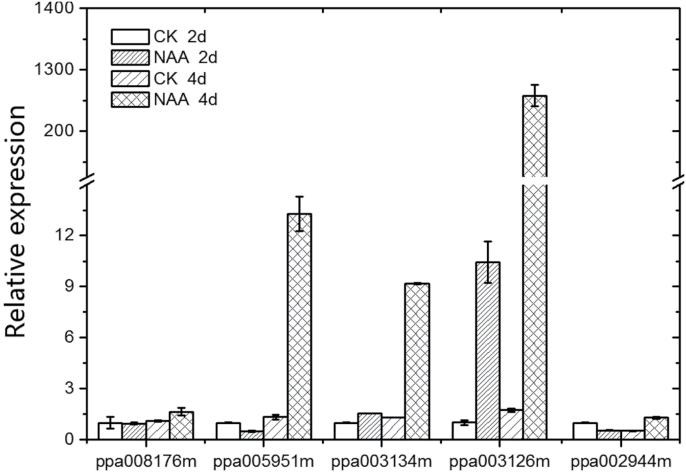
Effects of NAA on the expression of auxin-homeostasis-related genes in ‘Yumyeong’ fruit. Harvested mature fruits of ‘Yumyeong’ were treated with 1mM NAA each day. The steady-state levels were normalized to *actin*. Data are means ±SE of three individual experiments, each performed in triplicate. CK, control; NAA, NAA treatment; 2d or 4d, 2 d or 4 d, respectively.

The hierarchical cluster analysis shows that a couple of auxin-homeostasis-related genes showing a similar expression pattern to *PpACS1* in the DGE data for melting flesh peach (Fig. 2A). Previous studies have shown that a lot of auxin metabolic genes are auxin-response genes (Peer *et al.*, 2004; Terol *et al.*, 2006). Although IAA cannot be accumulated normally in stony hard peach cultivars, the auxin-homeostasis-related genes in SH flesh peaches should have a similar expression pattern (with exogenous auxin treatment) as the normal peach fruits. The different responses to exogenous auxin treatment of the candidates most likely reflect whether the gene’s expression is required for IAA accumulation or merely the result of a high IAA concentration. To understand the transcriptional changes in these candidate genes during the ripening stage of melting flesh peaches, the effect of exogenous auxin on the expression of auxin-homeostasis-related genes of stony hard peaches was examined. Mature fruit of ‘Yumyeong’ were treated with the synthetic auxin NAA. The expression levels of auxin-homeostasis-related genes that were found to cluster closely with *PpACS1* in the DGE analysis were examined (Fig. 3). The expression levels of these genes did not increase in control fruits; expression levels of the *ILL* gene (ppa005951m), the *PIN* gene (ppa002944m), and the two *GH3* genes (ppa003134m; ppa003126m) increased in NAA-treated ‘Yumyeong’ fruits after 2 d or 4 d of NAA treatment. By contrast, the expression of the *YUC* gene (ppa008176m) was not affected by exogenous NAA application to the fruits of ‘Yumyeong’ (Fig. 3).

### Structural features and polymorphic loci analysis of ppa008176m (*PpYUC11*)

**Fig. 4.**
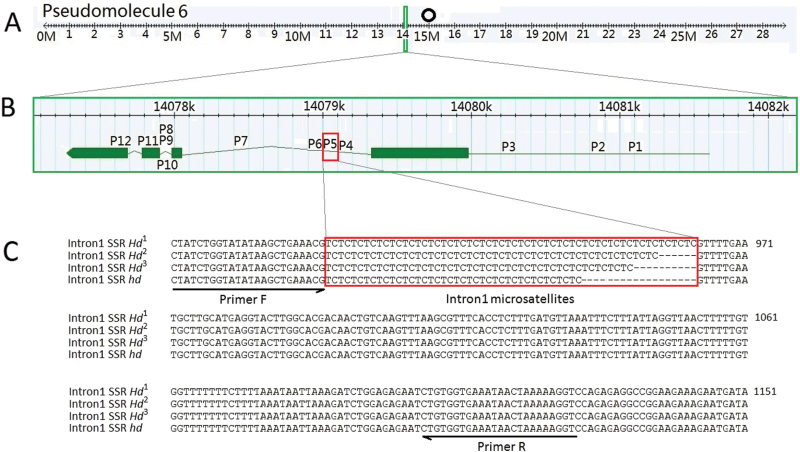
Graphical representation of chromosomal location, polymorphic loci, and the TC microsatellite loci of *PpYUC11*. (A) The chromosomal locations of *PpYUC11* are shown. The physical distance along the indicated pseudomolecule 6 is on the horizontal axis (Mb). The circle indicates the putative position of the centromeric regions. (B) Gene distribution of 12 polymorphic loci (P1–P12) of *PpYUC11*. (C) The microsatellites (*n*=29, 26, 24, or 20) in intron 1 of *PpYUC11*. The microsatellite locus is highlighted by red boxes, and the locations of primers used for polymorphic analysis are indicated by arrows. (This figure is available in colour at *JXB* online.)

Based on the observation that the expression patterns of ppa008176m (named *PpYUC11*, Fig. 7) and IAA concentrations of the two cultivars (Fig. 2) showed high levels of consistency, a detailed analysis was undertaken of the *PpYUC11* locus variations using the hypothesis that *PpYUC11* structural variation may cause differential expression of *PpYUC11* between the two cultivars. *PpYUC11*, contains four exons, located in pseudomolecule 6 and is near the centromeric regions in the peach genome sequence (Fig. 4A, B). The *PpYUC11* gene was re-sequenced from a set of homozygous cultivars. The analysed regions covered the entire transcribed region as well as the 1.7kb sequence upstream of the start codon (the promoter region). At least 12 polymorphic loci (Fig. 4B, P1–P12). were found in the *PpYUC11* gene using a combination of cloning and direct Sanger sequencing of PCR products: within the promoter region, a 25bp insertion/deletion (indel) and two SNPs are present in *PpYUC11* at positions -1068, -835, and -279bp of the published sequence of GenBank, respectively (Fig. 4B; see Supplementary Fig. S3A at *JXB* online); in intron 1, a 19bp indel, a TC dinucleotide microsatellite, and two SNPs are located at 852, 907, 910, and 1523bp, respectively (Fig. 4B; see Supplementary Fig. S3B at *JXB* online); in intron 2, an SNP, a single nucleotide indel, and an SNP are located at 2018, 2021, and 2040bp, respectively (Fig. 4B; see Supplementary Fig. S3B at *JXB* online); in addition, the coding region (exon 3 and exon 4) of the *PpYUC11* gene contains two nonsense mutation SNPs at 2104 and 2364bp (Fig. 4B; see Supplementary Fig. S3B at *JXB* online). Of these variations, the TC dinucleotide microsatellite is located just after the 19bp indel region at positions 907–964 of the GenBank reference sequence of intron 1 of *PpYUC11* gene (see Supplementary Fig. S3B at *JXB* online).

### *PpYUC11* allele distribution in peach germplasm

The sequencing of a PCR-amplified DNA fragment from a set of homozygous cultivars confirmed that the distribution of all *PpYUC11* variations can be found in both flesh types, with the exception of the TC dinucleotide microsatellite (see Supplementary Fig. S3B at *JXB* online). To investigate the relationship between the TC microsatellite genotype of *PpYUC11* and the phenotype of flesh texture (normal or stony hard), the distribution of the polymorphic loci was analysed in 36 normal and seven stony hard flesh cultivars (Table 1). To maximize genetic diversity, this set included nine American cultivars, 24 Chinese cultivars, nine Japanese cultivars, and one Korean cultivar.

**Table 1.** Allelic variants of *PpYUC11* as related to phenotype in various melting, non-melting, and stony hard flesh peach genotypes

Genotype	Phenotype	ppa008176m_SSR	Geographical origin
May fire	Melting	*Hd* ^*3*^ */Hd* ^*3*^	USA(B)
NJC19	Non-melting	*Hd* ^*3*^ */Hd* ^*3*^	USA(B)
NJC47	Non-melting	*Hd* ^*3*^ */Hd* ^*3*^	USA(B)
NJC77	Non-melting	*Hd* ^*3*^ */Hd* ^*3*^	USA(B)
NJC105	Non-melting	*Hd* ^*1*^ */Hd* ^*3*^	USA(B)
NJC112	Non-melting	*Hd* ^*3*^ */Hd* ^*3*^	USA(B)
Springtime	Melting	*Hd* ^*3*^ */Hd* ^*3*^	USA(B)
Spring Snow	Melting	*Hd* ^*3*^ */Hd* ^*3*^	USA(B)
Elberta	Melting	*Hd* ^*3*^ */Hd* ^*3*^	USA(B)
Okitsu	Melting	*Hd* ^*1*^ */hd*	Japan(B)
Azumo	Melting	*Hd* ^*1*^ */Hd* ^*3*^	Japan(B)
Hakuho	Melting	*Hd* ^*2*^ */Hd* ^*3*^	Japan(B)
Toobo	Melting	*Hd* ^*3*^ */Hd* ^*3*^	Japan(B)
Okubo	Melting	*Hd* ^*3*^ */hd*	Japan(B)
Kawanakajima Hakuto	Melting	*Hd* ^*1*^ */Hd* ^*1*^	Japan(B)
Hakuto	Melting	*Hd* ^*2*^ */hd*	Japan(B)
Matsumori	Melting	*Hd* ^*2*^ */Hd* ^*2*^	Japan(B)
Sunago Wase	Melting	*Hd* ^*2*^ */Hd* ^*2*^	Japan(B)
Hangzhou Zao Shui Mi	Melting	*Hd* ^*1*^ */Hd* ^*3*^	China(B)
Zao Feng Wang	Melting	*Hd* ^*1*^ */Hd* ^*1*^	China(B)
Bao Lu	Melting	*Hd* ^*2*^ */Hd* ^*2*^	China(B)
Yu Hua Lu	Melting	*Hd* ^*3*^ */hd*	China(B)
Li Chun	Melting	*Hd* ^*1*^ */Hd* ^*3*^	China(B)
Pan Tao Huang Hou	Melting	*Hd* ^*1*^ */Hd* ^*3*^	China(B)
Zao Lu Pan Tao	Melting	*Hd* ^*1*^ */Hd* ^*3*^	China(B)
Bai Hua	Melting	*Hd* ^*2*^ */hd*	China(L)
Chinese Cling	Melting	*Hd* ^*1*^ */Hd* ^*3*^	China(L)
CN 5	Melting	*Hd* ^*3*^ */hd*	China(B)
CN 9	Melting	*Hd* ^*1*^ */Hd* ^*1*^	China(B)
CN 8	Melting	*Hd* ^*1*^ */Hd* ^*3*^	China(B)
CN 13	Melting	*Hd* ^*3*^ */Hd* ^*3*^	China(B)
Goldhoney 1	Melting	*Hd* ^*1*^ */Hd* ^*3*^	China(B)
Goldhoney 3	Melting	*Hd* ^*3*^ */hd*	China(B)
Zhong Pan Tao 1	Melting	*Hd* ^*1*^ */Hd* ^*3*^	China(B)
Zhong Pan Tao 2	Melting	*Hd* ^*3*^ */hd*	China(B)
CP 5	Melting	*Hd* ^*3*^ */Hd* ^*3*^	China(B)
Yumyeong	Stony hard	*hd/hd*	Korea(B)
CN 16	Stony hard	*hd/hd*	China(B)
Xia Cui	Stony hard	*hd/hd*	China(B)
Shi Jia Zhuang	Stony hard	*hd/hd*	China(B)
Jing Yu	Stony hard	*hd/hd*	China(B)
Hua Yu	Stony hard	*hd/hd*	China(B)
Qing Wang	Stony hard	*hd/hd*	China(B)

Sequence variation in the SSR locus was analysed by means of microsatellite genotyping of PCR products. B, breeding material; L, landraces.

In our results, (TC)_*n*_ microsatellites were found in at least four different lengths (the repeat number ‘*n*’ of *Hd* ^*1*^, *Hd* ^*2*^, *Hd* ^*3*^, and *hd* are 29, 26, 24, and 20, respectively) in these peach cultivars (Fig. 4C). The diversity of microsatellite length variants exhibited dramatic differences between the 36 normal and the seven stony hard flesh peach cultivars. All seven stony hard peaches were homozygous for *hd*. By contrast, all of the normal peach cultivars had at least one *Hd* allele (*Hd* ^*1*^, *Hd* ^*2*^ or *Hd* ^*3*^). These results are summarized in Table 1.

### Transcriptional analysis of *PpYUC11* alleles

**Fig. 5.**
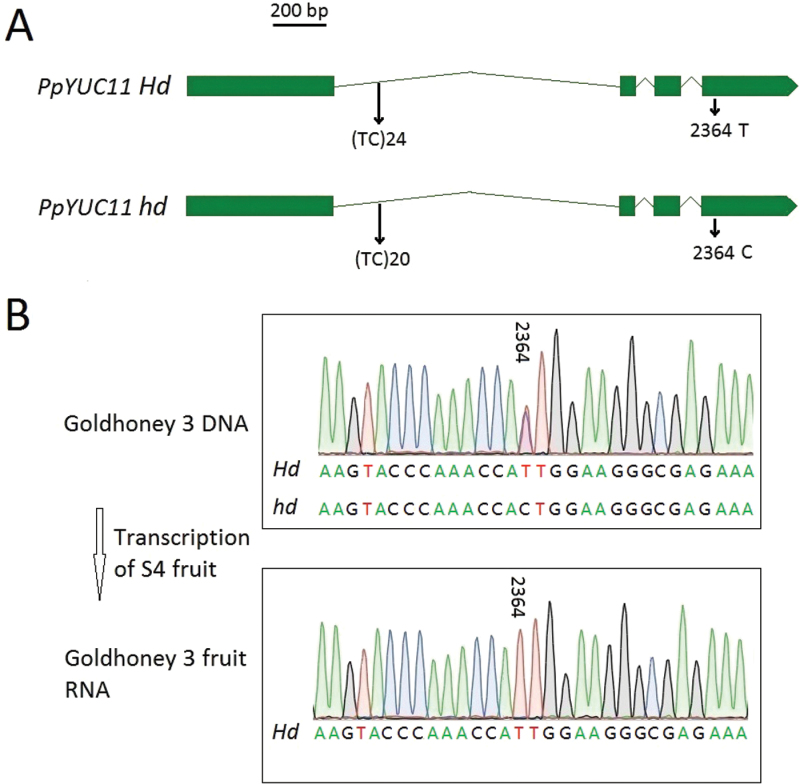
Transcriptional analysis of *PpYUC11* alleles of ‘Goldhoney 3’. (A) Polymorphic features of *PpYUC11* in ‘Goldhoney 3’ are shown. The *Hd* allele contains (TC)_24_ and 2364 T; the *hd* allele contains (TC)_20_ and 2364 C. (B) Direct Sanger sequencing of *PpYUC11* PCR products of DNA and stage S4 fruit cDNA (RNA) in heterozygous normal-fleshed peach ‘Goldhoney 3’ (*Hd*/*hd*). (This figure is available in colour at *JXB* online.)

The TC microsatellite identified to be responsible for the stony hard flesh phenotype appears to have an effect on transcriptional regulation of the gene. Direct Sanger sequencing was used to estimate the transcript levels of both alleles of *PpYUC11* in the mesocarp tissue of stage S4 in several peach varieties that are heterozygous at the TC microsatellite locus. For instance, sequencing of genomic DNA showed that ‘Goldhoney 3’ carries two different alleles at its *PpYUC11* locus: *Hd* (*n*=24), thymidine nucleotide (T) existing at position 2364bp, and *hd* with a cytidine nucleotide (C) at position 2364bp within the fourth exon of the *PpYUC11* gene (Fig. 5A). By sequencing the PCR products, it was found that, whereas this SNP is heterozygous in the genomic DNA of ‘Goldhoney 3’ (both C and T), by contrast, it is homozygous in the cDNA in the mesocarp of S4 fruit (Fig. 5B), supporting the hypothesis that the TC microsatellite is responsible for the transcriptional activity of *PpYUC11* and for IAA accumulation in ripening fruits and that *PpYUC11* is the candidate gene controlling the stony hard phenotype in peach.

### Co-ordinated variation of *PpYUC11* expression, IAA concentration, *PpACS1* expression, ethylene production, and fruit firmness in ripening peaches

**Fig. 6.**
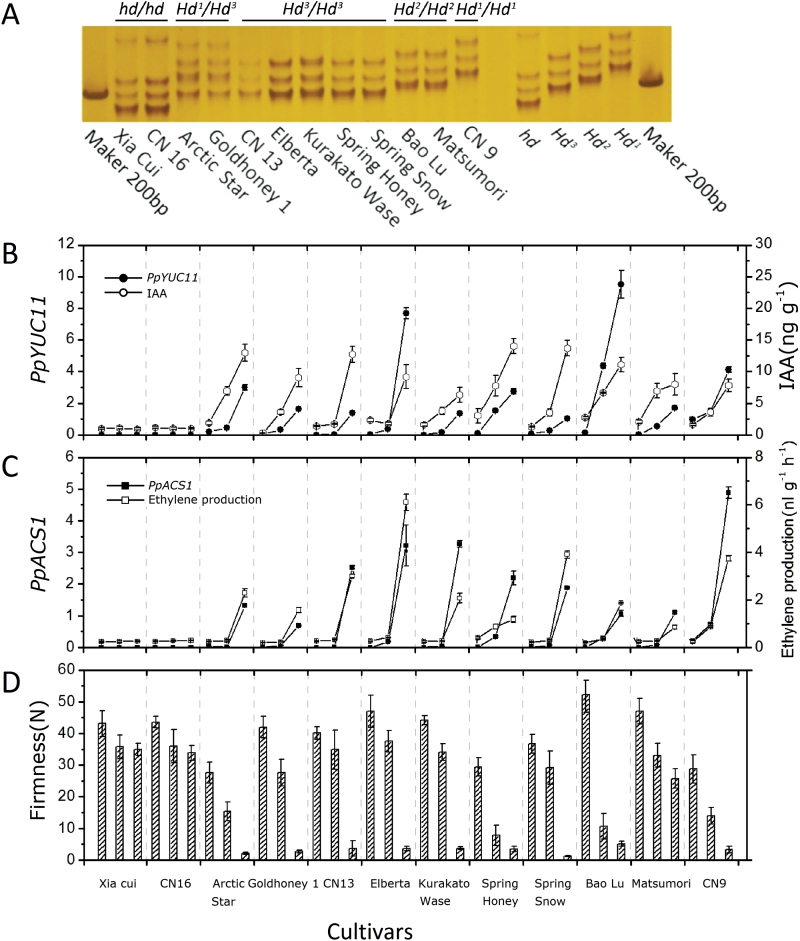
Co-ordinated variation of *PpYUC11* expression and IAA concentration, *PpACS1* expression and ethylene emission and fruit firmness in various ripening peaches. (A) The intron1 SSR genotype of *PpYUC11*in 12 peach cultivars. (B) Expression of *PpYUC11* and IAA contents, (C) expression of *PpACS1* and ethylene productions, and (D) fruit firmness in 12 peach cultivars. For each cultivar, three time points from stage S3 to S4 (from left to right) were selected. For qRT-PCR, normalization was made to the expression of the *actin* gene, and values are means of three technical replicates. For IAA concentrations or ethylene production, the results are the mean ±SE of measurements for at least three individual experiments; for flesh firmness, the results are the mean ±SE of measurements of at least five fruits. (This figure is available in colour at *JXB* online.)

A total of 12 peach cultivars with different genotypes in the *PpYUC11* SSR loci were selected and used to investigate the relationship between *PpYUC11* expression, IAA concentration, *PpACS1* expression, ethylene production, and fruit firmness in the mesocarp of fruits during the ripening transition stage (S3–S4). These cultivars belong to five genotypes: ‘Xia Cui’ and ‘CN16’ is *hd*/*hd*; ‘Arctic Star’ and ‘Goldhoney 1’ is *Hd* ^*1*^/*Hd* ^*3*^; ‘CN13’, ‘Elberta’, ‘Kurakato Wase’, ‘Spring Honey’, and ‘Spring Snow’ is *Hd* ^*3*^/*Hd* ^*3*^; ‘Bao Lu’ and ‘Matsumori’ is *Hd* ^*2*^/*Hd* ^*2*^; and ‘CN9’ is *Hd* ^*1*^/*Hd* ^*1*^ (Fig. 6A). For each cultivar, three samples from stage S3 to stage S4 were collected for further study. Overall, *PpYUC11* and *PpACS1* transcripts were highly expressed in the mesocarp of normal fleshed S4 fruits, but were either low or undetectable in the mesocarp of stony hard or S3 fruits (Fig. 6B, C). These expression profiles were accompanied by similar IAA content profiles or ethylene production in the mesocarp of these peach cultivars (Fig. 6B, C). Flesh firmness decreased towards harvest time along with ethylene emissions (Fig. 6D). These findings further confirmed that *PpYUC11* may regulate the IAA content in the mesocarp during the fruit ripening stage and may lead to different ethylene emissions and firmness of the peach fruits.

### YUCCA flavin monooxygenase family genes in peach

**Fig. 7.**
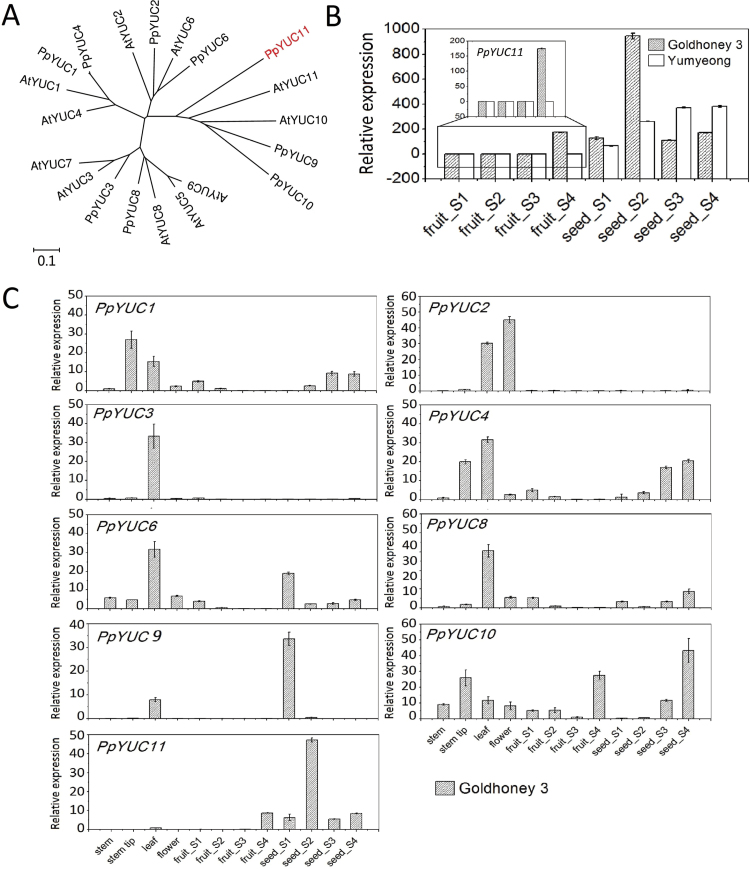
(A) Phylogenetic tree of *P. persica* and *Arabidopsis* YUC proteins. The phylogeny was constructed using the Neighbor–Joining method and a bootstrap test with 1 000 iterations using MEGA5 software, and alignments were generated with ClustalW. (B) Quantitative RT-PCR analysis of *PpYUC11* in fruit and seed of stage S1, S2, S3, and S4 of *P. persica* cv. ‘Goldhoney 3’ and ‘Yumyeong’. (C) Quantitative RT-PCR analysis of *YUC* genes in different tissues of *P. persica* cv. ‘Goldhoney 3’. The expression of nine peach *YUC* genes in stem, stem tips, leaf, flower, fruits of stage S1, S2, S3, and S4; and seeds of stage S1, S2, S3, and S4 were analysed. The steady-state levels were normalized to *actin*. Data are means ±SE of three individual experiments, each performed in triplicate. (This figure is available in colour at *JXB* online.)

The peach genome contains nine predicted *YUC*-like genes (Table 2; Fig. 7A). Based on the DGE analysis data, both *PpYUC10* (ppa007054m) and *PpYUC11* (ppa008176m) are enriched in the fruit ripening stage of ‘Goldhoney 3’, but *PpYUC11* cannot be detected in ‘Yumyeong’ fruit during the same period (Fig. 2; see Supplementary Table S3 at *JXB* online). The expression of the other seven *YUC*-like genes in this family (*PpYUC1*, *2*, *3*, *4*, *6*, *8*, and *9*) could not be detected in selected tissues of the two cultivars (see Supplementary Table S3 at *JXB* online).

**Table 2.** Characteristics of the *YUC* family in *Prunus persica*

Gene	GDR^*a*^	Chromosome	Position (Mb)	Length (mRNA)^*b*^	ESTs/cDNAs^*c*^
*PpYUC1*	ppa024927m	LG1	38.04	1230	0
*PpYUC2*	ppa022204m	LG7	20.66	1299	0
*PpYUC3*	ppa019979m	LG1	3.82	1275	0
*PpYUC4*	ppa025133m	LG8	1.88	591(partial)	0
*PpYUC6*	ppa005244m	LG1	37.13	1416	0
*PpYUC8*	ppa015832m	LG8	18.77	1272	0
*PpYUC9*	ppa024334m	LG8	1.15	1152	0
*PpYUC10*	ppa007054m	LG8	20.91	1288	HX869273.1
*PpYUC11*	ppa008176m	LG6	14.08	1248	DY643147.1;
					DY643060.1;
					FC862792.1;
					DY643612.1;
					DY644431.1;
					DY643303.1;
					DY645892.1

^*a*^ Accession numbers of the genes at the Genome Database for Rosaceae.

^*b*^ Length of the predicted mRNA in nucleotides.

^*c*^ Accession numbers of the ESTs/cDNAs at NCBI.

To uncover the roles for each *YUC* gene in plant development, the varied expression profiles of the nine *YUC* genes were analysed during vegetative and reproductive development of ‘Goldhoney 3’ using qRT-PCR (Fig. 7C). *PpYUC1*, the gene with the highest sequence identity to *PpYUC4* in peach (Fig. 7A), shows expression patterns similar to that of *PpYUC4* in the stem, leaf, flower, and seed (Fig. 7C). Among the nine predicted *YUC* genes, the expression of *PpYUC1*, *PpYUC4*, *PpYUC6*, *PpYUC8*, and *PpYUC10* could be detected in almost all 12 tissues, of which *PpYUC1*, *PpYUC4*, *PpYUC6*, and *PpYUC8* accumulated preferentially in the vegetative tissues (stem and leaf) and seeds, and young fruit. *PpYUC10* were also enriched in the developing fruit (stages S1 through S4) (Fig. 7B). For *PpYUC3*, however, mRNA accumulation was detected only in the leaves. *PpYUC9* showed expression only in the leaves and seeds of stage S1, whereas *PpYUC2* was detected only in the leaves and flowers. Interestingly, Pp*YUC11* was expressed at various stages in the seeds of ‘Goldhoney 3’ and ‘Yumyeong’ (Fig. 7B, C). However, only in the melting flesh fruit ‘Goldhoney 3’ did *PpYUC11* show obvious tissue-specific expression in S4 fruits (Fig. 7B). This result is consistent with the hypothesis that *PpYUC11* is responsible for IAA accumulation in the flesh of fruits at the ripening stage and is a candidate gene controlling the stony hard phenotype.

## Discussion

Fruit firmness is essential for efficient harvesting, handling, marketing, and storage (Byrne *et al.*, 2012). Due to the potential for the development of firmer peaches with tree-ripe flavour and longer storage life, the inheritance and physiological characteristics of a stony hard flesh texture have been extensively investigated for many years (Yoshida, 1976; Haji *et al*., 2003, 2005; Tatsuki *et al*., 2006, 2013; Begheldo *et al.*, 2008). Because IAA concentration may control ethylene production (Tatsuki *et al.*, 2013) and, in turn, the process of fruit softening in normal flesh peaches, our study investigated the expression patterns of auxin-homeostasis-related genes in the melting flesh peach ‘Goldhoney 3’ and stony hard peach ‘Yumyeong’ during the fruit ripening stage.

The distribution of auxin in plant organs is tightly controlled through synthesis, inactivation, and transport, and key regulatory steps and regulation mechanisms controlling auxin homeostasis have been extensively reviewed (Rosquete *et al.*, 2012; Korasick *et al.*, 2013; Sauer *et al.*, 2013). Differential accumulation patterns of IAA in ‘Goldhoney 3’ and ‘Yumyeong’ became evident from late stage S3, and reached a maximum at stage S4 III, by which time ‘Goldhoney 3’ fruits had accumulated approximately 10-fold more IAA than ‘Yumyeong’ fruits (Fig. 2D). Accordingly, the two cultivars showed strikingly different developmental expression patterns of auxin-homeostasis-related genes (Fig. 2A). However, the differential expression of several auxin-homeostasis-related genes appeared to be the effect, rather than the cause, of the differential IAA accumulation. For instance, in ‘Goldhoney 3’, the up-regulation of *GH3* (ppa003134m), *GH3* (ppa003126m), and *PIN* (ppa002944m) at late ripening stages appears to be caused by the high IAA levels in this phenotype because the expression levels of these genes increased in NAA-treated mature ‘Yumyeong’ fruit (Fig. 3), which is consistent with previous findings that *GH3* and *PIN* are auxin-responsive genes (Peer *et al.*, 2004; Terol *et al.*, 2006). Previous genetic studies demonstrated that the flavin monooxygenases of the YUC family are key enzymes in Trp-dependent auxin biosynthesis (Cheng *et al.*, 2007). In the present study, a YUCCA (YUC) flavin the monooxygenase gene, *PpYUC11*, showed an identical expression pattern to both *PpACS1* (Fig. 2C) and IAA accumulation (Fig. 2D) in both ‘Goldhoney 3’ and ‘Yumyeong’, suggesting that the low IAA concentrations at the late ripening stage of stony hard peaches may result from the suppressed expression of *PpYUC11*.

An examination of 43 peach genotypes allowed the identification of 12 polymorphisms within the *PpYUC11* gene as well as in the 1.7kb upstream flanking sequences: (i) a 25bp indel and two SNPs upstream of the start codon; (ii) a 19bp indel, a TC microsatellite, and two SNPs located in intron 1; (iii) an adenine nucleotide (A) indel and two SNPs located in intron 2; and (iv) two nonsense mutation SNPs in exon 3 and exon 4. Remarkably, the presence of a (TC)_20_ microsatellite (an allele named *hd*) appears to be associated with stony hard cultivars when present in a homozygous state, whereas normal-fleshed (M or NM) cultivars possess at least one other allele (*Hd* ^*1*^, *Hd* ^*2*^ or *Hd* ^*3*^) at the *PpYUC11* locus (Table 1). The transcriptional analysis of the two alleles of *PpYUC11* in the heterozygous variety ‘Goldhoney 3’ and the co-ordinated variation analysis of *PpYUC11* expression, IAA concentration, *PpACS1* expression, ethylene emission, and fruit firmness in ripening peaches lends support to the hypothesis that *PpYUC11* is a strong candidate gene for the control of the stony hard phenotype in peach.

Microsatellites, or simple sequence repeats (SSRs), represent a unique type of tandemly repeated genomic sequences (iterations of 1–6bp nucleotide motifs), which are abundantly distributed across genomes and demonstrate high levels of allele polymorphism (Chistiakov *et al.*, 2006). SSRs typically represent selectively neutral DNA markers. However, numerous lines of evidence have demonstrated that microsatellites are non-randomly distributed across protein coding regions, 3’-UTRs, 5’-UTRs, and introns because microsatellites can affect chromatin organization, the regulation of gene activity, recombination, DNA replication, the cell cycle, and the mismatch repair (MMR) system (see the review by Li *et al.*, 2004). For instance, a tetranucleotide polymorphic microsatellite located in the first intron of the tyrosine hydroxylase gene regulates its gene expression (Meloni *et al.*, 1998). In the present study, both the (TC)_24_ and (TC)_20_ forms of a TC dinucleotide polymorphic microsatellite localized in the first intron of *PpYUC11* were found heterozygously in the genomic DNA of ‘Goldhoney 3’, but Sanger sequencing of the mesocarp tissue cDNA during ripening recovered only the transcript containing the (TC)_24_ microsatellite (*Hd*), indicating that the intronic TC microsatellite of *PpYUC11* may regulate the gene expression during ripening and lead to IAA accumulation. However, the causal relationship between the TC microsatellite polymorphisms and the transcriptional level remains to be formally proved. As a whole, our results strongly suggest that the (TC)_20_ microsatellite (*hd*) interferes with *PpYUC11* transcription.

Quantitative RT-PCR analysis revealed that *PpYUC11* is preferentially expressed in the seeds of both flesh types (‘Goldhoney 3’ and ‘Yumyeong’) during all developmental stages but is only expressed in the mesocarp of stage S4 in the melting flesh cultivar ‘Goldhoney 3’ (Fig. 7B), suggesting that there is another regulatory element, perhaps a transcription factor, that interacts with the *hd* locus to control the transcription of *PpYUC11* in both seed and flesh during different stages of fruit development. Depending upon the existence or non-existence of this transcription factor in flesh, *PpYUC11* will be selectively expressed in stage S4; by contrast, *PpYUC11* will be constitutively expressed in other tissues, such as seed. Based on this study’s results, both *PpYUC10* and *PpYUC11* are enriched in the fruit ripening stage of ‘Goldhoney 3’, but *PpYUC11* cannot be detected in ‘Yumyeong’ fruit in the same period (Fig. 2; see Supplementary Table S3 at *JXB* online), suggesting that *PpYUC10* is responsible for the biosynthesis of the remaining basic IAA levels in ‘Yumyeong’ and that *PpYUC11* is responsible for the high concentration of IAA in ‘Glodhoney 3’.

Auxin has long been considered a negative regulator for fruit ripening, as the content of auxin is low at the initiation of ripening (Nitsch, 1952). The decline of auxin content before the initiation of ripening has been suggested to be a prior condition for fruit ripening (Given *et al.*, 1988; Chen *et al.*, 1999; Purgatto *et al.*, 2002; Böttcher *et al.*, 2010) because the application of exogenous auxins has been shown to delay the ripening of fruits (Cohen, 1996; Purgatto *et al.*, 2002; Fabbroni *et al.*, 2006). It has been suggested that the low auxin levels in the ripening fruit tissues may be controlled by ripening-associated GH3 genes, which are involved in auxin conjugation (Böttcher *et al.*, 2011; Schaffer *et al.*, 2013). It is interesting that, in contrast to most fruit species, in peach fruits, the concentration of auxin increases suddenly just before ripening and coincides with climacteric ethylene production (Tatsuki *et al.*, 2013). Auxin induces the expression of genes encoding ACC synthase (Tatsuki *et al.*, 2013). The observations from the current study indicate that, in peach, the flavin monooxygenase gene *PpYUC11* may regulate the concentration of IAA at the late ripening stage. A higher expression level and stronger up-regulation of *PpYUC11* at the transition stage from maturation to the ripening stage (S3 to S4 III) were correlated with IAA concentration and *PpACS1* activation in ‘Goldhoney 3’ and another ten normal-fleshed cultivars (Figs 2, 6). By contrast, it is possible that, in ‘Yumyeong’, ‘Xia cui’, and ‘CN16’, less efficient auxin biosynthesis in the mesocarp due to the very low level of *PpYUC11* gene expression may contribute to the reduced IAA level and the stony hard phenotype (Figs 2, 6). The contrasting expression profiles in melting and stony hard flesh peaches of a YUCCA flavin monooxygenase (*PpYUC11*) during peach fruit ripening are consistent with the hypothesis that a low IAA concentration in ripening stony hard peaches could result from the suppression of IAA biosynthesis, the acceleration of IAA inactivation and degradation, the transition from free IAA to IAA storage forms, or the inhibition of IAA transport from biosynthetic tissues (Tatsuki *et al.*, 2013).

Peaches originated and diversified from China, and Chinese peach germplasm has had a great impact on breeding research in other countries (Li *et al.*, 2013). After the introduction of ‘Chinese Cling’ (‘Shanghai Suimitsuto’) as parents in the early 20th century, Japan selected out ‘Hakuto’ (Yamamoto *et al.*, 2003; Xu *et al.*, 2006) and the USA released the well-known cultivar ‘Elberta’. Both ‘Hakuto’ and ‘Elberta’, selected from seedlings of ‘Chinese Cling’, were extensively used as parents for further breeding of modern cultivars (Scorza *et al.*, 1985; Aranzana *et al.*, 2012). ‘Hakuto’ was reported to be heterozygous (*Hd*/*hd*) for the stony hard gene (Yoshida, 1976), accordingly, ‘Hakuto’ is heterozygous (*Hd* ^*2*^/*hd*) in the intronic TC microsatellite locus in our study (Table 1). The genotype of ‘Elberta’ at the TC microsatellite locus is *Hd* ^*3*^/*Hd* ^*3*^, which explains the lack of any stony hard peach cultivars in modern American history (Goffreda *et al.*, 1999). In our study, the genotype of ‘Chinese Cling’ was found to be *Hd* ^*1*^/*Hd* ^*3*^, a result that is in conflict with the genotype of the progeny ‘Hakuto’ (*Hd* ^*2*^/*hd*). It has been suggested that ‘Chinese Cling’ was not a single but, rather, a group of cultivars (Wang and Zhuang, 2001); if so, only a subset of this group of cultivars may have carried the *hd* allele, and the ‘Chinese Cling’ that was tested may not have been from this subset. Similar examples are ‘Jing Yu’ (*hd*/*hd*), a well-known stony hard cultivar from China, selected from among the crosses of ‘Okubo’ (*Hd*/*hd*)×’Okitsu’ (*Hd*/*hd*) and ‘Qin Wang’ (*hd*/*hd*, stony hard), which is a new peach cultivar selected from the seedlings of ‘Okubo’ (*Hd*/*hd*). The pedigrees of all these cultivars are consistent with the hypothesis that the intronic TC microsatellite of *PpYUC11* is the *hd* locus.

Stony hard peaches are characterized by the absence of both ethylene production and softening in mature fruits and are expected to be used as a genetic source for breeding new table peaches (Haji, 2014). However, stony hard fruits are often very difficult to distinguish from NM or very firm, unripe M phenotypes in the field, thereby making reliable selection difficult (Bassi and Monet, 2008; Byrne *et al.*, 2012). Genotyping the TC microsatellite of *PpYUC11* can now be used for the marker-assisted breeding of new cultivars of the stony hard flesh type.

## Conclusions

In this study, DGE analysis of auxin-homeostasis-related genes in melting flesh and stony hard peaches during fruit ripening has been reported. This strategy allowed several auxin-homeostasis-related genes to be identifed showing a similar expression pattern to the ACC synthetase gene *PpACS1*. The expression pattern, predicted function, and tissue distribution make *PpYUC11* an excellent candidate gene for stony hard type peaches. It is proposed that a TC microsatellite in its first intron is responsible for regulating its gene expression in the mesocarp. In addition, the marker developed on this sequence polymorphism provides a convenient molecular tool with which to discriminate normal/stony hard flesh cultivars or individuals in breeding populations. Our data indicate that a TC microsatellite in *PpYUC11* is the molecular basis of stony hard flesh peaches, and our results provide a basis for detailing breeding programmes for peach fruit texture improvement.

## Supplementary data

Supplementary data can be found at *JXB* online.

Supplementary Fig. S1. Schematic representation of the auxin homeostasis pathway in normal or stony hard flesh peaches.

Supplementary Fig. S2. qRT-PCR validation of 11 differentially expressed genes as detected by DGE analysis.

Supplementary Fig. S3. Alignment of the *PpYUC11* in melting and stony hard flesh cultivars.

Supplementary Table S1. qRT-PCR primers used in this study.

Supplementary Table S2. The full list of the normalized expression of 28 702 peach genes detected by DGE analysis in eight libraries.

Supplementary Table S3. The full list of 51 auxin-homeostasis-related genes and four ripening-related genes detected by DGE analysis in eight libraries.

Supplementary Data
