# Supplementary material for: PpYUC11, a strong candidate gene for the stony hard phenotype in peach (Prunus persica L. Batsch), participates in IAA biosynthesis during fruit ripening
Source: J Exp Bot. 2015 Aug 24;66(22):7031–44. doi: 10.1093/jxb/erv400 (PMC4765781; doi:10.1093/jxb/erv400)
Supplement: Supplementary Data [file supp_66_22_7031__index.html]

 PpYUC11, a strong candidate gene for the stony hard phenotype in peach (Prunus persica L. Batsch), participates in IAA biosynthesis during fruit ripening — Supplementary Data 

# *PpYUC11*, a strong candidate gene for the stony hard phenotype in peach (*Prunus persica* L. Batsch), participates in IAA biosynthesis during fruit ripening

## Supplementary Data

Data files

- Supplementary\_figure\_1\_3.pdf - Supplementary Data
- Supplementary\_table\_S1.xlsx - Supplementary Data
- Supplementary\_table\_S2.xlsx - Supplementary Data
- Supplementary\_table\_S3.xlsx - Supplementary Data
