# Supplementary material for: PpYUC11, a strong candidate gene for the stony hard phenotype in peach (Prunus persica L. Batsch), participates in IAA biosynthesis during fruit ripening
Source: J Exp Bot. 2015 Aug 24;66(22):7031–44. doi: 10.1093/jxb/erv400 (PMC4765781; doi:10.1093/jxb/erv400)
Supplement: Supplementary Data [file supp_erv400_Supplementary_figure_1_3.pdf]

PpYUC11, a strong candidate gene for the stony hard phenotype in peach (Prunus persica L. Batsch), participates in IAA biosynthesis during fruit ripening

Lei Pan, Wenfang Zeng, Liang Niu, Zhenhua Lu, Hui Liu, Guochao Cui, Yunqin Zhu, Jinfang Chu, Weiping Li, Weichao Fang, Zuguo Cai, Guohuai Li, and Zhiqiang Wang

Supplemental Figures

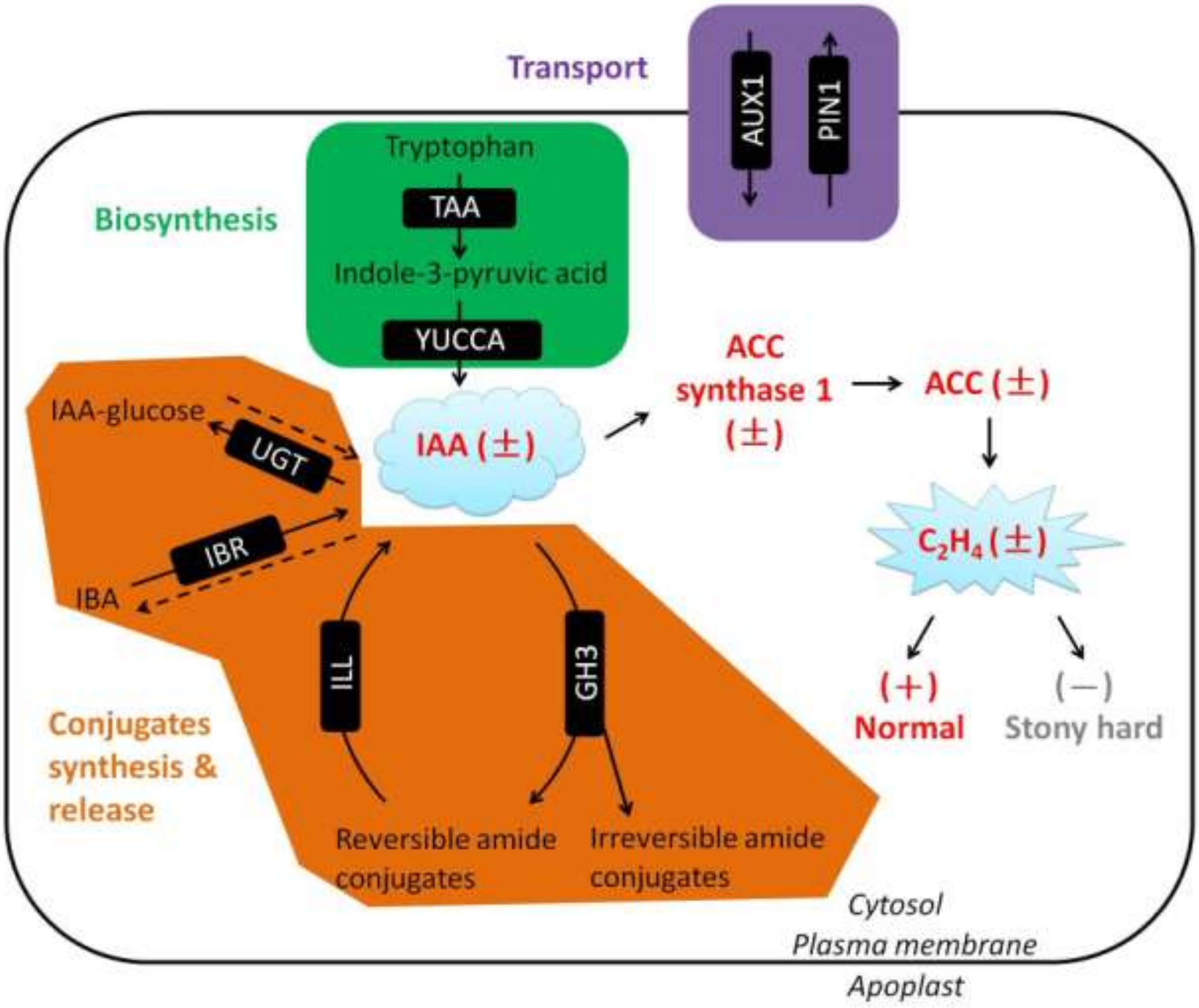

Fig. S1. Schematic representation of the auxin homeostasis pathway in normal or stony hard flesh peaches. The pathways of IAA de novo biosynthesis, conjugate conversion, and auxin transport according to Sauer et al. (2013) are shown. Auxin resistant 1 (AUX1), Gretchen Hagen 3 (GH3), indole-3-acetic acid (IAA), indole butyric acid (IBA), IAA-leucine resistant 1-like (ILL), IBA resistant (IBR), UDP-glucosyl transferase (UGT), PIN-formed (PIN), Tryptophan aminotransferase of Arabidopsis (TAA), YUCCA flavin monooxygenase (YUC).

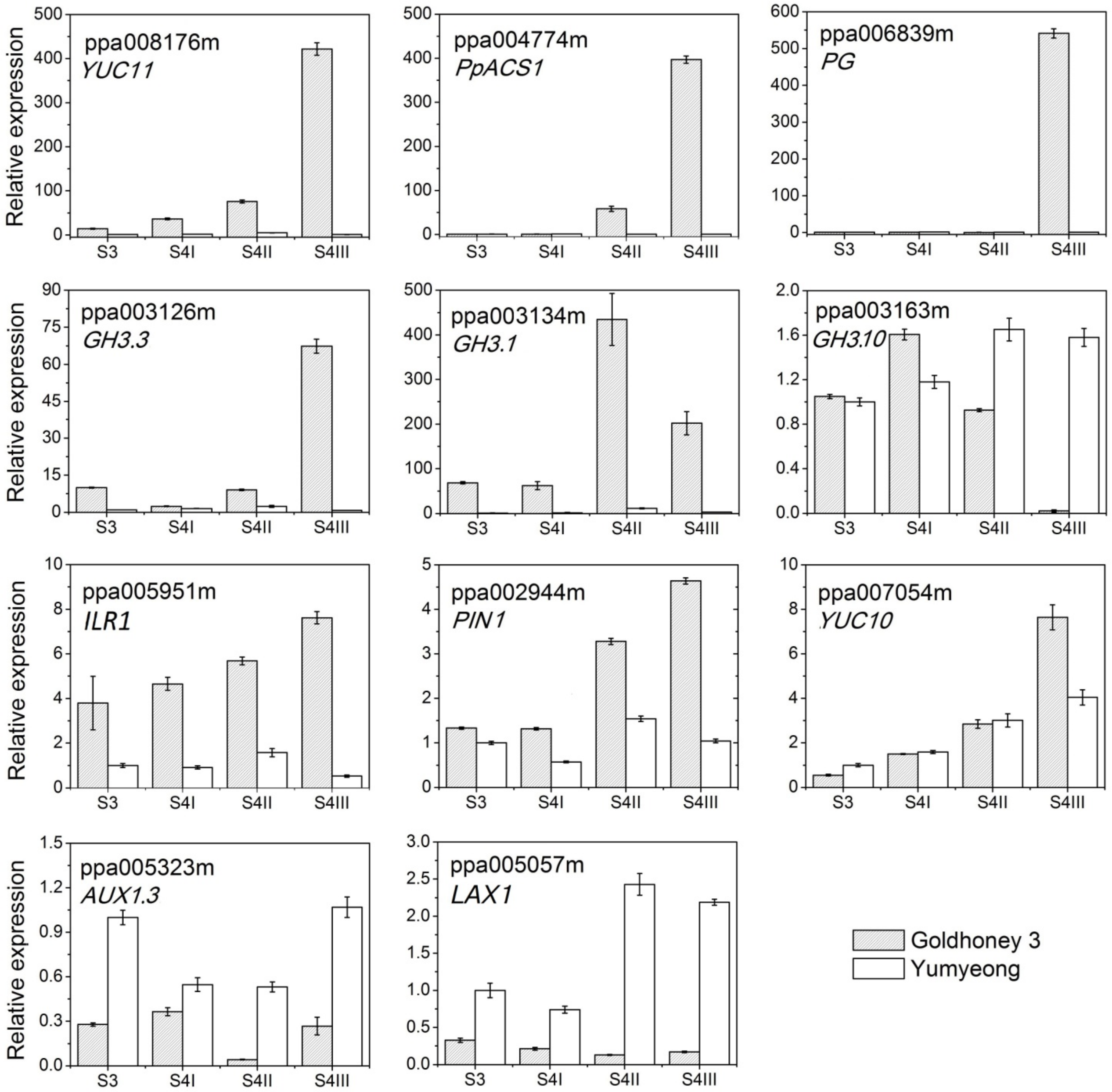

Fig. S2. qRT-PCR validation of 11 differentially expressed genes as detected by the DGE analysis.

(A)

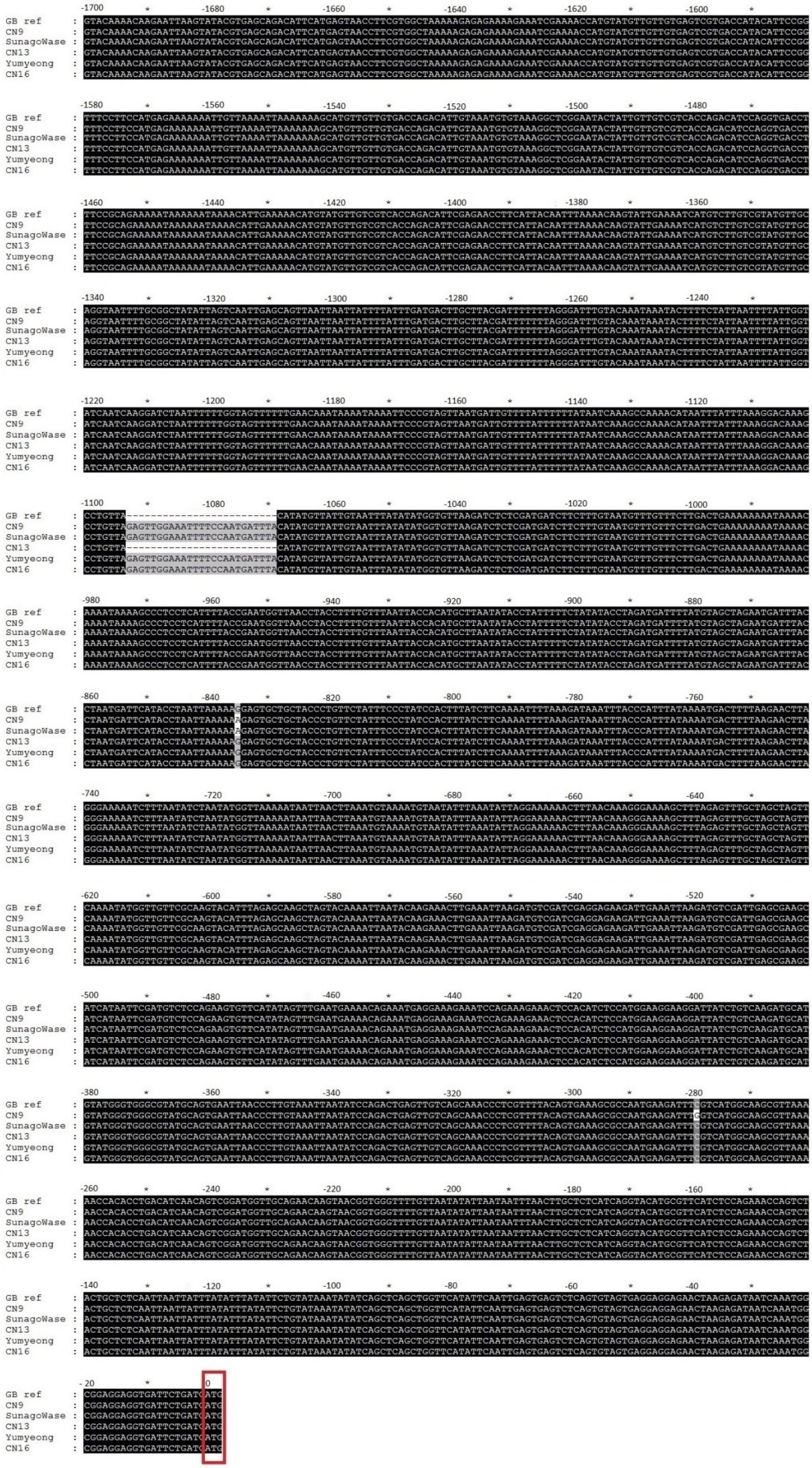

1

(B)

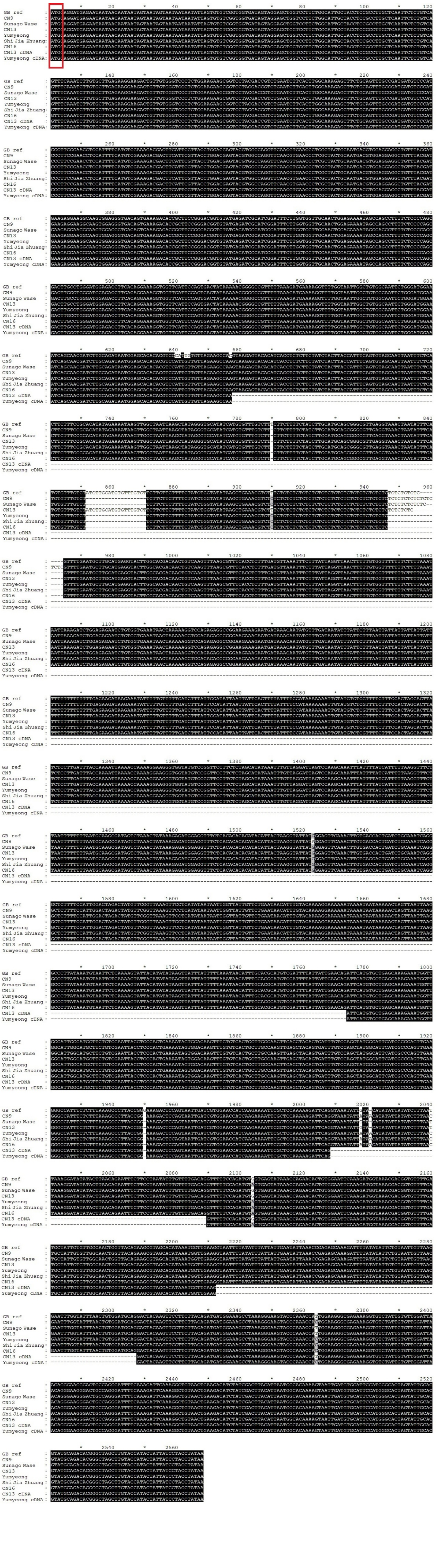

Fig. S3. Alignment of the *PpYUC11* in melting and stony hard flesh cultivars. (A) Alignment of the promoter region of the *PpYUC11* gene spanning positions -1700-0 of DNA in melting flesh ‘CN9’, ‘Sunago Wase’, ‘CN13’ and stony hard flesh ‘Yumyeong’, ‘CN16’. (B) Alignment of the translated region of the *PpYUC11* gene spanning positions 0-2568 of DNA in melting flesh ‘CN9’, ‘Sunago Wase’, ‘CN13’; stony hard flesh ‘Yumyeong’, ‘CN16’, ‘Shi Jia Zhuang’; and cDNA of ‘CN13’, ‘Yumyeong’.
